# Easy-handling minimum mass laser target scaffold based on sub-millimeter air bubble -An example of laser plasma extreme ultraviolet generation-

**DOI:** 10.1038/s41598-020-62858-3

**Published:** 2020-04-03

**Authors:** Christopher S. A. Musgrave, Shuntaro Shoji, Keiji Nagai

**Affiliations:** 1 Laboratory for Chemical and Life Sciences Institute of Innovative Research, Tokyo Institute of Technology R1-26 Suzukake-dai, Midori-ku, Yokohama, 226-8503 Japan; 2 Present Address: Centre of Micro/Nano Manufacturing Technology (MNMT-Dublin), University College Dublin, D14 YH57 Dublin, Ireland; 3 School of Chemical Science and Engineering, Tokyo Institute of Technology, R1-26, Suzukake-dai, Midori-ku, Yokohama, 226-8503 Japan

**Keywords:** Chemistry, Energy science and technology, Materials science, Nanoscience and technology, Optics and photonics

## Abstract

Low density materials can control plasma properties of laser absorption, which can enhance quantum beam generation. The recent practical extreme ultraviolet light (EUV) is the first industrial example of laser plasma source with low density targets. Here we propose an easy-handling target source based on a hollow sub-millimeter microcapsule fabricated from polyelectrolyte cationic and anionic surfactant on air bubbles. The lightweight microcapsules acted as a scaffold for surface coating by tin (IV) oxide nanoparticles (22–48%), and then dried. As a proof of concept study, the microcapsules were ablated with a Nd:YAG laser (7.1 × 10^10^ W/cm^2^, 1 ns) to generate 13.5 nm EUV relatively directed to laser incidence. The laser conversion efficiency (CE) at 13.5 nm 2% bandwidth from the tin-coated microcapsule (0.8%) was competitive compared with bulk tin (1%). We propose that microcapsule aggregates could be utilized as a potential small scale/compact EUV source, and future quantum beam sources by changing the coating to other elements.

## Introduction

Low density materials are a type of material that are significantly lower in density than the mother source. For example, polystyrene has a density of ~1.0 g/cm^3^, and corresponding very low density polystyrene can be as light as 0.03 g/cm^3^ ^1,2^. Low density materials are versatile; used in many applications such as tissue engineering^3^, high surface area matrix^4^, and widely throughout laser plasma experiments. Within laser plasma experiments, critical electron density of plasma is a key parameter to determine the absorption of laser resulting high energy density state, generating of quantum beam^5,6^. Ultralow density less than the critical density, typically ~1 mg/cm^3^, is desired to control the plasma character^7,8^. Recently, practical applications of extreme ultraviolet (EUV) include lithography for production of <7 nm integrated circuits^9^.

EUV light sources require a high laser conversion efficiency (CE %) from laser light, and robustness of the reflective Mo/Si optics over long operating periods^10^. At present, the most reliable EUV light sources utilize liquid tin droplets for ablation by a double pulse laser scheme due to the high power (250 W) at 13.5 nm^11^. High repetition rates (∼100 kHz) can be achieved using liquid tin droplets. However, the double pulse scheme struggles to control the droplet expansion dynamics where the droplet was illuminated by a prepulse to expand just microseconds before the main laser pulse^11–13^. The EUV collector durability also remains a problem. Finally, liquid tin requires high temperatures to melt (232 °C). This not ideal for practical handling, especially when new generations of laser quantum beam sources are designed. Easy-handling of high-repetition and high CE laser target without prepulse illumination is an crucial factor^14,15^.

Overcoming the limitations of liquid tin dynamics control can be very advantageous in generating EUV. Synthesis of well-defined low density tin targets have a merit for supporting a wide range of materials consisting of various elements, exact shape, pore size, density etc^16–20^. Plasma generated from low density materials or nanostructured targets have a reduced opacity, increasing the CE as the plasma becomes less dense^6,18–26^. Moreover, the flexibility of materials science is exciting for quantum beam sources; the desired wavelength of light can be selected based on specific elements supported by a low density scaffold. For example, some elements supported by low density scaffolds include gold^17^, copper^27^, vanadium^27^ and titanium^28^ for x-ray generation. For EUV generation, an extremely low density tin (10^19^ atoms/cm^3^) is required, we aim here to define a new concept of low density targets for this purpose.

A technique derived from a layer-by-layer (LbL) fabrication method^29,30^ has previously been explored to produce low density materials for EUV light generation^20^. One LbL method produces a polyelectrolyte microcapsule that can be coated with tin nanoparticles or electrodeposited onto the surface. The now tin-coated microcapsule can be ablated to generate 13.5 nm EUV. The benefit of LbL methods is that the raw materials are low-cost, mechanically stable, and the coating of nanoparticles are not restricted to tin. However, in those EUV experiments a solid particle template was used in the formation of the LbL capsule, which was then removed before laser irradiation^20,31^. The process of removing the solid template would not be favorable in situations were a higher volume of targets which are required to be processed quickly such as high-repetition laser experiments. Therefore, we were motivated to develop a LbL microcapsule based on a substrate free^32,33^ or gas template in a similar manner to a previously published structure^34^. A gas template can be simply incorporated into the LbL capsule without supercritical fluid drying process, and can be treated as a laser target. Furthermore, a simple fabrication process could be scaled-up into a higher volume production for consideration in high-repetition EUV generation, or extend to other quantum beam generation due to a freedom of doping materials.

Here, we show the fabrication of LbL polyelectrolyte microcapsules from a gas (air) template. The microcapsules were coated with tin oxide (SnO_2_) nanoparticles, with alternating layers of tin oxide and polyelectrolytes to increase tin content. The capsules were ablated using a 1064 nm Nd:YAG (7.1 × 10^10^ W/cm^2^, 1 ns, 60 μm spot size) laser to generate 13.5 nm EUV light. The CE at 13.5 nm 2% bandwidth was estimated for bulk tin and the microcapsules.

**Figure 1:**
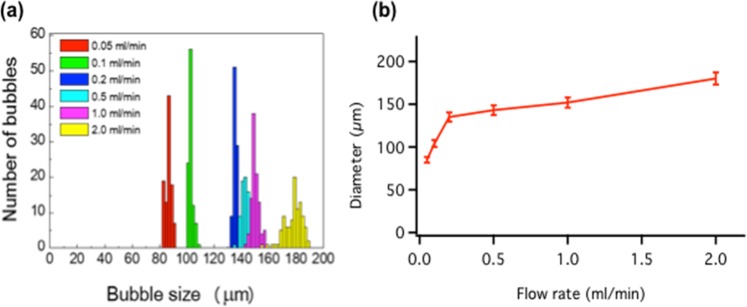
Polyelectrolyte microcapsule diameter control using a 7.2 × 10^−2^ M DTAB solution with varying flow rate.

We used an adapted version of a LbL fabrication technique to produce polyelectrolyte microcapsules composed of poly(sodium 4-styrene-sulfonate) (PSS) and poly(allylamine hydrochloride) (PAH)^20,29–34^. We used a gas tight syringe and pump to form the initial microcapsule. The air template was encapsulated with either dodecyltrimethylammoniumbromide (DTAB) or poly(vinyl alcohol) (PVA). Furthermore, using an air template meant one less stage of processing was required compared with a solid particle template. The flow rate of the syringe pump was chosen between 0.05–2.0 ml/min to control the microcapsule diameter (Figs. 1 and S1). The wet microcapsules diameters were monodisperse at the flow rates we tested, with a diameter variation of 4% at each flow rate. The maximum rate of microcapsule production was about 200 Hz, which also correlated with other parameters such as capsule diameter. The ability to select the microcapsule diameter is hugely flexible compared with microcapsules based on the particle template, which is limited to 10 μm variation due to collapse of the capsule during template decomposition. Control over the capsule diameter has the advantage of customizable diameter depending on the application; for example tuning the diameter to different laser spot sizes. The microcapusles were then coated in a tin oxide nanoparticle solution, and dried for characterization.

Inductively coupled plasma atomic emission spectroscopy (ICP-AES) measured 4.2 × 10^−9^ g of Sn was present in the microcapsule core layers, which was equivalent to 2.1 × 10^13^ Sn atoms (Supplementary Information). Our microcapsules were unique compared with previous methodologies in that additional layers of alternating PAH/SnO_2_ layers were applied over the PSS/PAH core layers. This increased the overall tin content with only a monolayer of PAH between each SnO_2_ layer. We performed this in order to capitalize on alternating ionic charge between the SnO_2_ nanoparticle solution (−) and the PAH (+) to form these additional stablizing layers.

**Figure 2:**
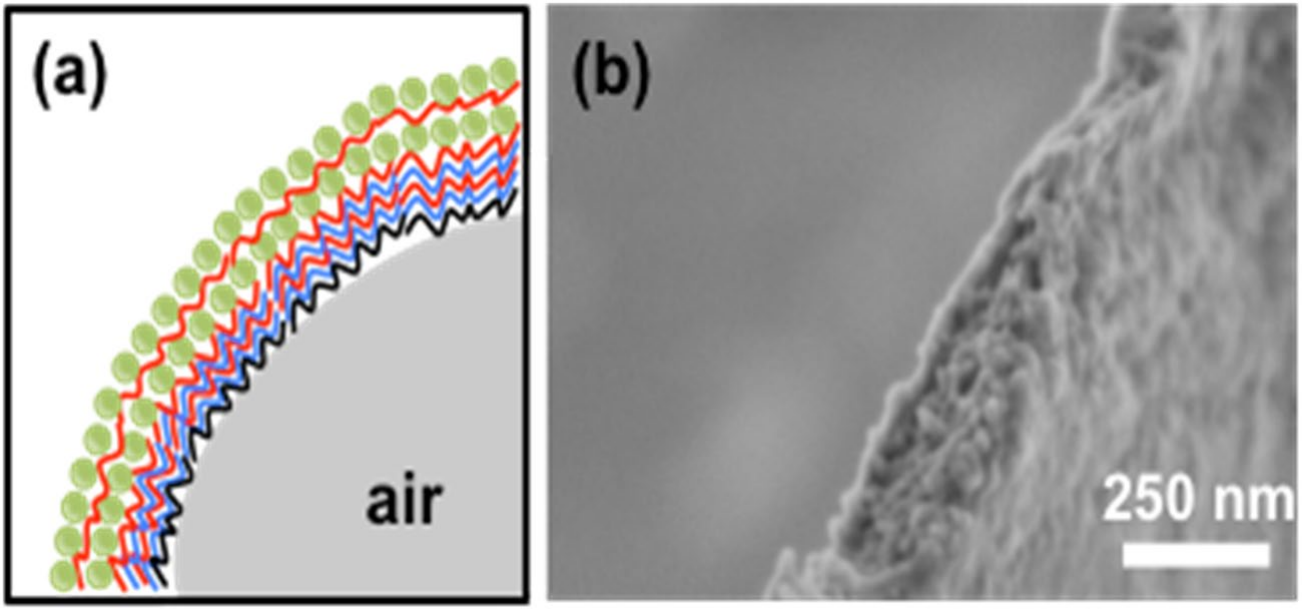
A schematic of the microcapsule composition (left), and a cross-sectional FE-SEM image (right). The schematic represents two layers of SnO_2_ coating whereas the fabricated microcapsules contained 3- or 6-layers. The FE-SEM image shows a microcapsule wall cross-section composed of PVA[PSS/PAH]_3_[SnO_2_/PAH]_2_SnO_2_.

**Figure 3:**
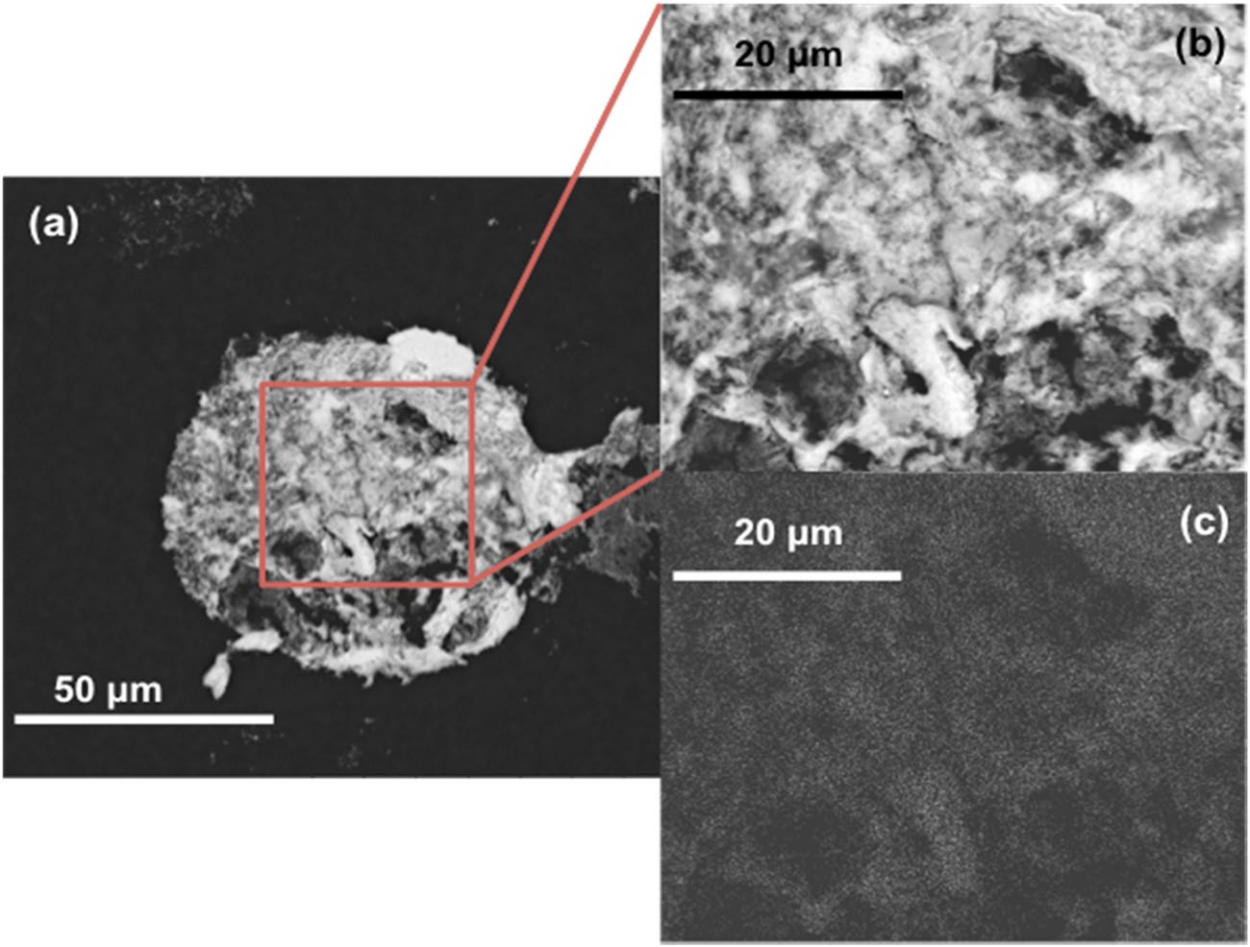
SEM images of a dry tin oxide coated microcapsule target (**a**) and (**b**), and corresponding EDS mapping of elemental tin (**c**). The lighter specks (**c**) correspond to the tin oxide nanoparticles. The images are of a PVA[PSS/PAH]_3_[SnO_2_/PAH]_2_SnO_2_ microcapsule with 28% tin content by mass.

Field emission scanning electron microscopy (FE-SEM) (Fig. 2(b)) revealed that the thickness of the microcapsule walls were around 180 nm. The cross-section almost resolved the individual layers of the microcapsule, particularly the final SnO_2_ layer. The schematic in Fig. 2(a) is not to scale, but shows the LbL composition of each polyelectrolyte ion from the DTAB or PVA core (black), PSS (blue) and PAH (red) layers followed by coating with SnO_2_ nanoparticles. Figure 3 shows another SEM image from the topview and Fig. 3(b) is a zoom-in image of a part of Fig. 3(a). Energy dispersive X-ray spectroscopy (EDS) measured 22–48% by mass Sn coating on the surface of the microcapsules (Fig. 3(c)). The SnO_2_ coating was relatively well-distributed across the surface, even in cases were 22% Sn was measured. This meant there was no severe variations expected in the wall thickness (approx. 15 nm SnO_2_ nanoparticle size from transmission electron mircoscopy (TEM)).

**Figure 4:**
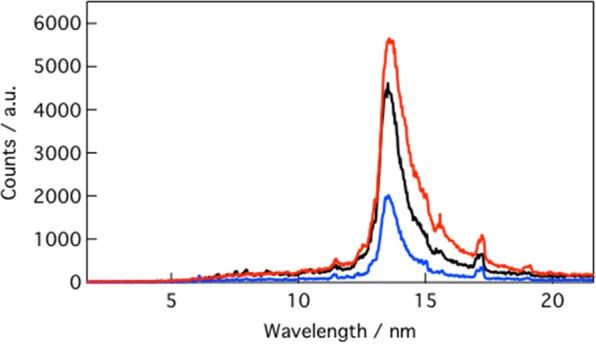
EUV emission spectra of bulk tin (black), 6-layer SnO_2_ microcapsule (red) and 3-layer SnO_2_ microcapsule (blue) ablated using a 7.1 × 10^10^ W/cm^2^ 1 ns pulse.

As a case study for the effectiveness of the microcapsule scaffold, we generated 13.5 nm EUV light using a 1064 nm Nd:YAG laser. The capured EUV spectra can be seen in Fig. 4. ICP-AES measured that there were 2.1 × 10^13^ Sn atoms present in a microcapsule. This was comparable to the minimum mass calculated elsewhere where 7.4 × 10^13^–1.5 × 10^14^ atoms was suitable for EUV generation^35,36^. Thus, we expected a strong EUV emission at 13.5 nm associated with ablation of tin within the microcapsules. The EUV spectra were characterized as a strong unresolved transmission array (UTA) emission around 13.5 nm arising from tin transmissions between Sn^8+^ and Sn^21+^ ^35^. The in-band CE at 13.5 nm 2% bandwidth was estimated at 0.8% for the 6-layer SnO_2_ microcapsules using a previously described technique^37^. This was lower than the ideal CE (3%) for bulk tin^38^, but comparable to that of bulk tin (1%) at the same laser condition. The lower tin efficiency would be due to a lateral expansion loss of ablation plume for the present smaller laser spot (60 μm) and shorter pulse duration (1 ns) than the cases of previous reports (for example >100 μm, >6 ns)^39,40^. Such lateral expansion would exhibit less opacity effect as re-absorption of 13.5 nm light due to the small plume. This resulted in a sharper spectrum in comparison the previous spectrum produced by Nd-YAG laser irradiation, while larger spot size and long pulse duration gave so-called corona plasma which re-absorbs 13.5 nm light from the radiation region^35^.

The 6-layer CE of 0.8% was in contrast to 0.4% CE of the 3-layer SnO_2_ microcapsules. Therefore our microcapsule preparation method was justified; the additional layers of SnO_2_ increased the tin content sufficiently for a strong EUV emission. The EUV spectra suggested that the laser was over-penetrating the top layers of SnO_2_/PAH resulting in ablation of more SnO_2_. Thus, more SnO_2_ was ablated for a strong emission in the 6-layered microcapsules compared with the 3-layered target, resulting in a higher CE. Further improvements to the microcapsule SnO_2_ coverage and laser parameters could yield a higher CE than bulk Sn.

The microcapsules exhibited relatively directed EUV emission to laser incidence in comparison to bulk tin as seen angular distribution data (Fig. S4 and Table S1 in ESI). The EUV emission from the 3-layered target was more directed than that from the 6-layered one, suggesting a localized emission point at the front of laser incident^41^ due to the present minimum mass tin.

**Figure 5:**
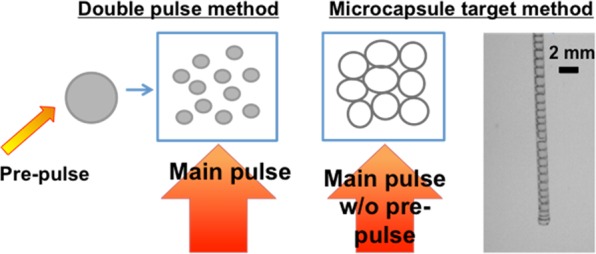
Scheme comparing current EUVL double-pulse method (left) to the proposed microcapsule aggregate target (right). The aggregate would represent a “mist” in a similar manner to current methods requiring two pulses. A high-speed camera image shows some progress at Tokyo Tech to date. The microcapsules can be produced in a large volume, which could be used for continuous target supply.

In practice, the microcapsules had a tendency to coalesce into a larger aggregate during fabrication and transportation for laser shots. This meant that 13.5 nm EUV was generated from a microcapsule aggregate. Aggregated capsules are not a serious issue as the synthesis method could be adapted for higher repetition EUV generation. Firstly, the microcapsule diameter was both monodisperse and customizable (~80–180 µm). Secondly, the facile preparation method could be adapted into a continuous fabrication process and supply of the microcapsule aggregates to the laser focus position. Lastly, the microcapsule structure is such that only one driving pulse would be required to generate EUV. A pre-pulse would be redundant as the microscapsule is already a low density structure/aggregate. The microcapsules would be analogous to the presently used double-pulse method to create a tin mist from liquid tin before ablation by the main driving pulse^11–13^. Fig. 5 shows how the microcapsule aggregate represents the double-pulse method. We are actively interested in developing such a higher-repetition microcapsule aggregate target delivery system.

However, with EUV entering high volume manufacturing (HVM) stages, there are several points to overcome. Firstly, issues with microcapsule transportation to the focal spot precisely and frequently. A ~50 m/s speed of target delivery is required, while such high speed with high accuracy has been studied by target fabricators^42^. The second issue for these targets and HVM is the frequency of capsule production, which was about 200 Hz in the present air bubble. We would need to bundle and integrate ~100 fabrication devices as a simultaneous operation including target injection to the focal spot. The other point is problems the carbon debris from the capsule if it is not fully removed by the magnetic field shield^35^. These points would be faced on high-repetition >1 kHz, and EUV HVM. On the other hand, relatively low-repetition (<100 Hz) does not prove so difficult to construct the present concept of laser quantum beam source with a single fabrication device.

Finally, in this paper we used tin oxide as the coating material on the surface of the polyelectrolyte scaffold. The intent was to show the fabrication and practical usage of a LbL scaffold microcapsule for metallic nanoparticles. In this case 13.5 nm EUV light was generated. However, it is feasible to use many other nanoparticles to generate other quantum beams, and wavelengths of EUV light. For example, Gd (6 nm) is of interest as a beyond EUV source^43,44^, which can be found in nanoparticle form. A detailed review on general uses, including surface coatings, of microcapusles have been discussed elsewhere^5^.

In summary, EUV light is becoming increasingly important in today’s world and becomes more expensive as the high volume manufacturing of integrated circuits is realized. However, double pulse illumination is one of several issues that cannot be ignored much longer. To this end we have prepared a lightweight, stable layer-by-layer (LbL) tin oxide coated polyelectrolyte microcapsule scaffold. The facile synthesis route utilized a gas template core to fabricate the monodisperse microcapsules. To prove the effectiveness of the capsules, we generated 13.5 nm EUV light using a Nd:YAG laser (7.1 × 10^10^ W/cm^2^). A maximum CE of 0.8% for the LbL microcapsules vs 1% of bulk tin at 13.5 nm 2% bandwidth. A scheme for an aggregate microcapsule target was proposed, akin to the currently used double-pulse scheme. Such an easy-handling low density target contributes to construction of a compact EUV source better suited for imaging^45^ or surface modification^46^ rather than HVM EUV source. Finally, we highlight that other wavelengths of light could be generated by changing the scaffold nanoparticle coating.

## Experimental Section

### Materials

All materials were used as received unless stated otherwise. Poly(vinyl alcohol) (PVA) (Mw 1,500–1,800, Wako chemicals), Poly(allylamine hydrochloride) (PAH) (Mw 17,500, Aldrich), poly(sodium 4-styrene-sulfonate) (PSS) (Mw 70,000, Aldrich), deionized water, dodecyltrimethylammoniumbromide (DTAB) (Tokyo Chemical Industry), sodium chloride (Wako chemicals), tin (IV) oxide nanoparticles (<100 nm (BET), Aldrich).  The particle size characterization is shown in Figs S2 and S3.

The nanoparticle size was checked using by transmission electron microscopy (TEM) (TEM7000, Hitachi). The average particle size was 15 nm, with a distribution between 10–50 nm.

### Layer-by-layer microcapsule fabrication and characterization

All of procedures are done in a laminar flow cabinet equipped with HEPA filter. The microcapsules were prepared by using a gas-tight syringe (Hamilton) attached to a syringe pump. We fabricated syringe needles with an inner diameter of either 27 μm or 108 μm, and inserted into a solution of 7.2 × 10^−2^ M DTAB or 3 wt% PVA. Once the PVA or DTAB core bubbles were produced, they were washed with water to remove excess PVA or DTAB solution. A process of adding the electrolytes was applied to form the LbL microcapsules in the manner of PSS coating (1 mg/ml), washing by water, PAH coating (1 mg/ml), washing, and repeated 3 times (with each layer composing of one coating of PSS and PAH each).

The microcapsules were then immersed in a tin oxide nanoparticle solution (1 mg/ml) for 1–2 minutes, and then coated again with PAH (1 mg/ml). This was repeated either twice or five times, with a final coating of tin oxide nanoparticles to give the completed microcapsules. The capsules were then dried on a glass substrate (Asahi) overnight.

Inductively coupled plasma atomic emission spectrometry (ICP-AES) (PerkinElmer ELAN DRC-e) was performed on tin-oxide coated microcapsules. The mass of tin was obtained, allowing calculation of the number of tin atoms present in the microcapsules. Details are shown in Supplementary Information.

A Field Emission Scanning Electron Microscope (FE-SEM) (Hitachi SU8020) operating in a low accelerating voltage mode (1 kV) imaged the cross-section of the dry microcapsule walls. Microcapsules were sputter-coated with several nm of platinum to improve conductivity of the electron beam. The metal coating also protected the polyelectrolyte capsule from any damage caused by the electron beam.

A mini-SEM (Chip Hua, TE3000) was used to perform Energy-Dispersive X-ray Spectroscopy (EDS) measurements. The corresponding SEM images were obtained at an accelerating voltage of 15 kV in secondary electron mode. Microcapsules were not sputter-coated with metal, as this would have interfered with the EDS measurements.

### Laser irradiation conditions

A 1064 nm Nd:YAG laser (2 mJ, 1 ns, L11038–01, Hamamatsu Photonics) with a spot size of 60 μm full width half maximum (7.1 × 10^10^ W/cm^2^) was used to irradiate the microcapsules. A charged couple device (CCD) camera (D0920-BN, Tokyo Instruments) was used to obtain the EUV spectra at 45 degree angle with respect to the angle of laser ablation inccidence. Bulk tin was used a reference material (100 μm thick, Nilaco, Japan). The in-band CE at 13.5 nm 2% bandwidth was estimated for bulk Sn, and the microcapsule targets using a previously described method using phosphor imaging plates and a calorimeter^38^. The vacuum chamber operated at a pressure in the region of 10^−5^ Torr.

## Supplementary information

Supplementary Information.
