# Supplementary material for: Easy-handling minimum mass laser target scaffold based on sub-millimeter air bubble -An example of laser plasma extreme ultraviolet generation-
Source: Sci Rep. 2020 Apr 3;10:5906. doi: 10.1038/s41598-020-62858-3 (PMC7125169; doi:10.1038/s41598-020-62858-3)
Supplement: Supplementary file 1 — Supplementary Information. [file 41598_2020_62858_MOESM1_ESM.pdf]

Electronic Supporting Information

Easy-handling minimum mass laser target scaffold based on sub-millimeter air bubble -An example of laser plasma extreme ultraviolet generation-

Christopher S A Musgrave[1], Shuntaro Shoji[2], and Keiji Nagai[1,2]

1 Laboratory for Chemical and Life Sciences, Institute of Innovative Research, Tokyo Institute of Technology, R1-26 Suzukake-dai, Midori-ku, Yokohama 226-8503, Japan.

2 School of Chemical Science and Engineering, Tokyo Institute of Technology.

Corresponding author: nagai.k.ae@m.titech.ac.jp

Contents

Materials page 2- 4

Characterization of tin content by ICP-AES page 5

Characterization of EUV emission by Imaging plate analysis of bulk tin and tin with bubble scaffold page 6-7

References page 8

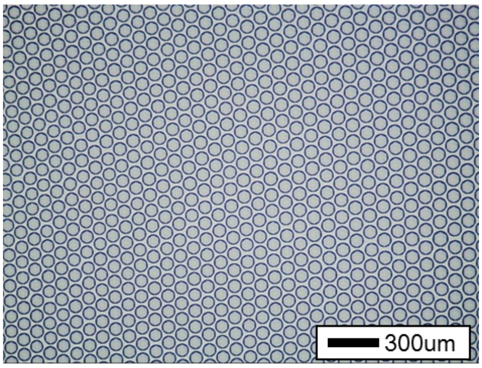

Fig S1. Optical image of monodisperse microcapsules for the flow rate of 0.5 mL/min as shown in Figure 1.

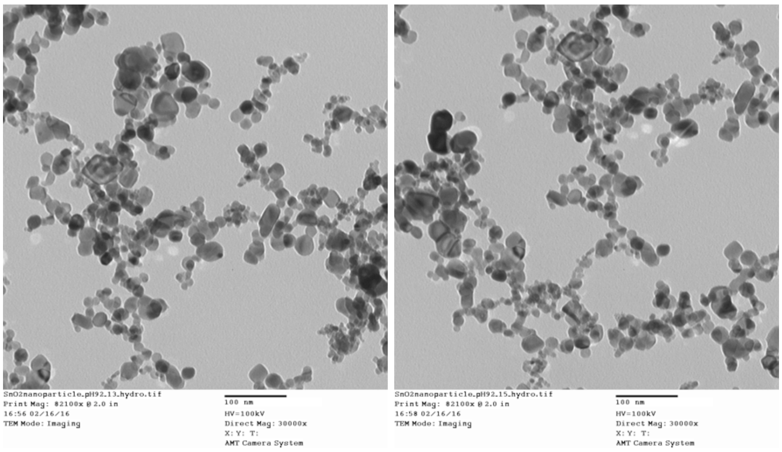

Fig S2. TEM images of the SnO_2 nanoparticles.

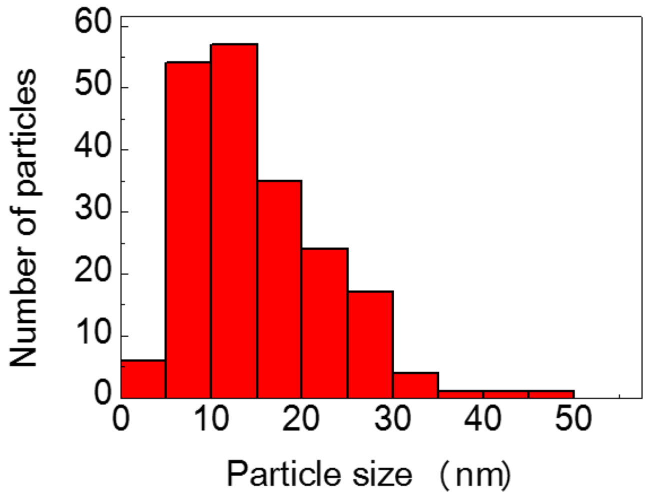

Fig S3. Histogram distribution of the nanoparticle size measured from TEM.

ICP-AES

ICP-AES was performed using a Perkin Elmar ELAN-DRC-es ICP apparatus. The experiments used the tin-coated bubble scaffolds to measure the tin content quantitatively. Several tin solutions with accurately known concentrations (ppm) were used calibration of the tin quantity. A solution of 500 ppm tin (II) sulfate ($SnSO_4$) was prepared using 10mg $SnSO_4$ added to 20mL of a 10% concentrated sulfuric acid (H_2SO_4) and hydrochloric acid (HCl) and stirred at 70°C for 3 hours. A 50 ppm $SnSO_4$ solution was prepared by adding 200 mL 36% HCl to the 500 ppm solution and stirred at 70°C for 1 hour. A solution of at 5 ppm $SnSO_4$ was finally prepared diluting the 50 ppm solution 10-fold. Substrates with tin (II) oxide (SnO_2) were prepared by combing SnO_2 nanoparticles in a solution of 10% H_2SO_4 with 1 mg/mL PAH to the amount of 2.5 and 0.25 ppm. Dliution of the 2.5 ppm solution to 0.25 ppm was performed by addition of 10% conc. mixture of HCl and H_2SO_4. Liquid samples were then used to calibrate the ICP apparatus before analyzing the tin-coated bubble scaffolds.

Imaging plate analysis of bulk tin and bubble scaffolds

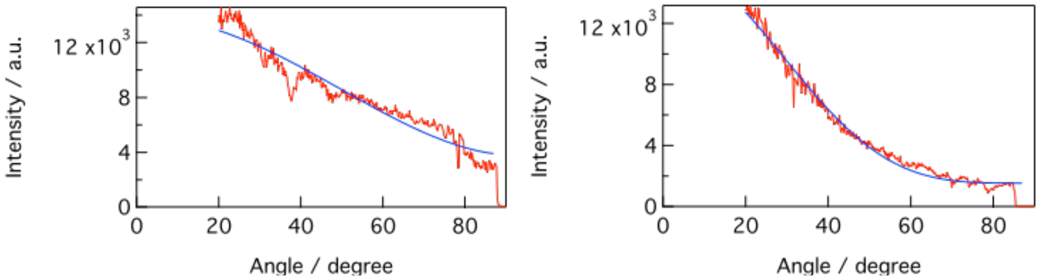

Fig S4. Imaging plate fitting curves for a 6-layered (left) and 3-layers bubble scaffold (right). Areas not covered by the Zr filter can be seen in the bottom right of each image, highlighting the effect of the unfiltered light on the IP. The angles covered are between 20-90 degrees.

An imaging plate (IP) (BAS-TR, Fujifilm, Japan), composed of a $BaFBr:Eu^{2+}$ film, was set between 20-90 degrees with respect to the incidence laser and target. The experimental set-up, apparatus and methodology was similar to a previously described experiment.[1] The plate had a Zr filter set (100nm thick, NTT-AT, Japan) between the laser target and imaging plate to filter longer wavelengths of light (>20nm) that would otherwise be recorded by the IP. The generated light from target ablation excites meta-stable 'F-centers' on the IP, which can then photoluminesce to relaxed state using a reading device (Rigaku, RAXIA-Di, Japan). Thus an intensity vs angle image can be obtained for each target across the angles the plate was set. The intensity data was analysed using a fitting function described as follows and in more detail elsewhere.[2]

$$I(\theta) = [I(0) - I(\theta)]\cos^{x}\theta + I(90) \qquad (1)$$

, where $I(\theta)$ is the intensity at θ degree. The fitting equation parameter $\cos^{x}\theta$ provides information on the direction of EUV emission.[2]

The angular distribution is required to obtain conversion efficiency (CE) from laser energy as estimated by eq (2).

$$CE = \int_0^{2\pi}\int_0^{\pi/2} I(\theta)Ecal(45)/I(45)\,d\theta\,d\Omega \qquad (2)$$

, where $E(45)$, $I(45)$, and LE, are energy at 45°, imaging plate signal at 45°, and irradiated laser energy, respectively. The $E(45)$ value was registered by the calorimeter to be 4.0 x 10^{-5} J/sr according to the same condition as the previous research for tin targets [2], where the ratio of inband (10-20 nm) per out band (longer than 20 nm) emission was 1:1.8.[3]

The fitting gave the parameters as Table 1.

Table S1. EUV emission at 13.5nm 2% bandwidth and IP fitting values for tin, 6-layer and 3-layer microcapsule targets.

Material	2% bandwidth ratio at 13.5nm (between 10-20nm)	*x* value for $\cos^x \theta$	[I(90)-(-I(0)]/I(0)
Bulk Sn	0.123	0.52	1.12
6-layer SnO_2	0.155	1.71	1.30
3-layer SnO_2	0.175	4.1	1.12

References

1. Musgrave, C. *et al*. High-space resolution imaging plate analysis of extreme ultraviolet (EUV) light from tin laser-produced plasmas. *Rev. Sci. Instrum*., **88**, 033506 (2017).

2. Nagai, K. *et al*. Angular distribution control of extreme ultraviolet radiation from laser-produced plasma by manipulating the nanostructure of low-density SnO_2 targets. *Appl. Phys. Lett*., **88**, 094102 (2006).

3. Yuseph, M. *et al*. Dynamics of laser-produced Sn microplasmas for a high-brightness extreme ultraviolet light source. *Appl. Phys. Lett*., **98**, 201501 (2011).
